# The effectiveness of a golf injury prevention program (GRIPP intervention) compared to the usual warm-up in Dutch golfers: protocol design of a randomized controlled trial

**DOI:** 10.1186/s13102-022-00511-4

**Published:** 2022-07-26

**Authors:** S. Gladdines, A. L. von Gerhardt, E. Verhagen, A. Beumer, D. Eygendaal, GRIPP 9 study collaborative

**Affiliations:** 1 Department of Orthopaedic Surgery, Amphia Hospital, Molengracht 21, PO box 90158, 4818CK Breda, The Netherlands; 2 Department of Orthopaedics and Sports Medicine, Erasmus University Medical Center, Rotterdam, The Netherlands; 3 Department of Orthopaedic Surgery, Amsterdam Movement Sciences, Amsterdam UMC, University of Amsterdam, Meibergdreef 9, Amsterdam, The Netherlands; 4 Academic Center for Evidence-Based Sports Medicine (ACES), Amsterdam, The Netherlands; 5 Amsterdam Collaboration on Health and Safety in Sports (ACHSS), AMC/VUmc IOC Research Center, Amsterdam, Netherlands; 6 Amsterdam Collaboration for Health and Safety in Sports, Department of Public and Occupational Health, Amsterdam Movement Sciences, Amsterdam University Medical Centers, Location VU University Medical Center, Amsterdam, The Netherlands; 7 Coronel Institute of Occupational Health, Department of Public and Occupational Health, Amsterdam University Medical Centers, Amsterdam, The Netherlands

**Keywords:** Golf, Injury, Prevention, Golf swing, Warming-up

## Abstract

**Background:**

Sixty million golfers around the world play golf. Golf injuries are most frequently located in the spine, elbow, wrist, hand and shoulder. Those injuries are often seen in golfers with more playing hours and suboptimal swing biomechanics, resulting in overuse injuries. Golfers who do not perform a warm-up or do not warm-up appropriately are more likely to report an injury than those who do. There are several ways to warm-up. It is unclear, which warm-up is most useful for a golfer to perform. Moreover, there is currently no evidence for the effectiveness of a warm-up program for golf injury prevention. We previously have developed the Golf Related Injury Prevention Program (GRIPP) intervention using the Knowledge Transfer Scheme (KTS). We aim to evaluate the effect of the GRIPP intervention on golf-related injuries. The hypothesis is that the GRIPP intervention program will reduce the number of golf-related injuries.

**Methods and design:**

The GRIPP study is a two-armed randomized controlled trial. Twenty-eight golf clubs with 11 golfers per club will be randomly allocated to the intervention or control group. The intervention group will perform the GRIPP intervention program, and the control group will perform their warm-up as usual. The GRIPP intervention is conducted with the Knowledge Transfer Scheme framework, which is a systematic process to develop an intervention. The intervention consists of 6 exercises with a maximum total of 10 min. The primary outcome is the overall prevalence (%) of golf injuries measured with the Oslo Sports Trauma Research Center (OSTRC-H) questions on health problems every fortnight. The secondary outcome measures will be exposure to golf and compliance to the intervention program.

**Discussion:**

In other sports warm-up prevention programs are effective in reducing the risk of injuries. There are no randomized trials on golf injury prevention. Therefore, an individual unsupervised golf athlete intervention program is conducted which reflects the daily practice of predominantly unsupervised exposure of amateur golfers.

**Trial registration:**

The trial is retrospectively (28 October 2021) registered at the Dutch Trial Register: NL9847 (https://trialsearch.who.int).

## Background

Worldwide 60 million people play golf in 206 countries [1, 2]. In the Netherlands, The Dutch Golf Federation is the third-largest federation of ball games societies after soccer and tennis [3]. As individuals age, participation in previously accessible leisure activities can be compromised through diminished capabilities and negative societal expectations [4]. Golf is, however, a popular sport for older adults and an important source of physical activity [5, 6]. The population of golfers in Europe consists of 84% of golfers older than 40 years [7].

**Fig. 1:**
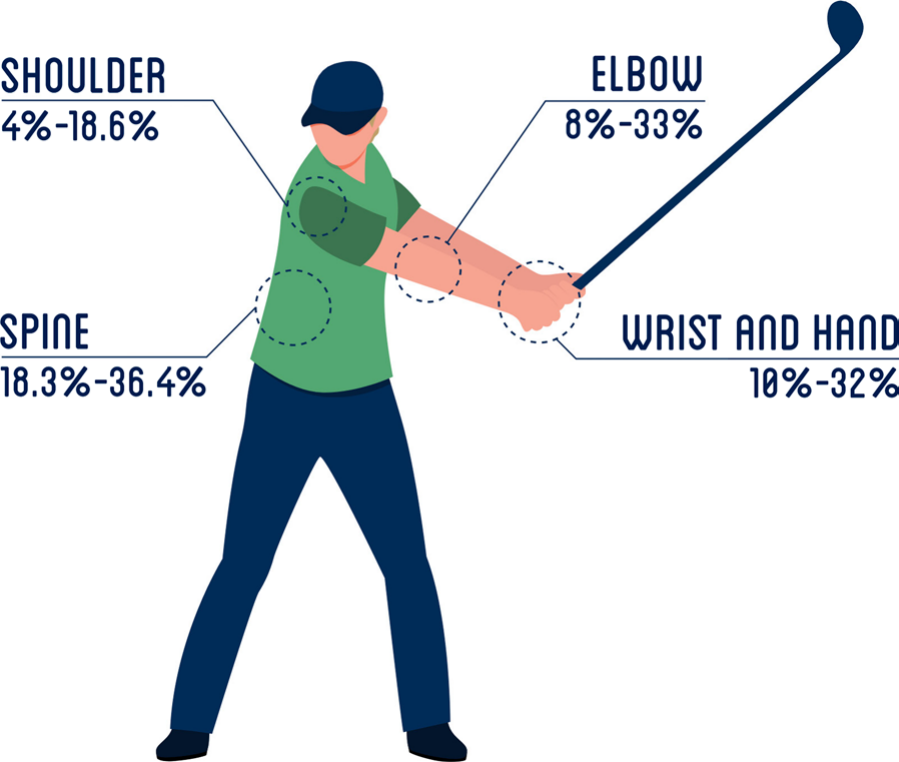
Distribution of injuries in the upper limb and spine in recreational golfers

Despite the known contribution of sports to health and well-being, sports participation declines in older age. Sports play an important role in older age and contribute to better health and well-being for some people [8]. As in all sports, injuries do occur. Figure 1 shows the injury distribution in recreational golfers. Those injuries are most frequently located in the spine, elbow, wrist and hand, and shoulder [6]. Golf overuse injuries are related to a higher intensity of playing and suboptimal swing biomechanics [6, 9].

Various studies in populations of recreational golfers reported golf-related injuries with an incidence up to 36.5% [10–14]. Recreational golfers are 3.2 (odds ratio) times more likely to report an injury when they do not perform a warm-up [15]. It is unclear which type of warm-up could be most useful for a golfer to prevent injuries. In other sports, such as volleyball, handball, and baseball, effective warm-up prevention programs were previously assessed, positively impacting injury reduction [16–18].

The golf swing is considered one of the most difficult movements in sports. To perform a golf swing, there is a powerful action required with rapid force generation [19]. Therefore, large rotational forces are transferred to the golf ball with compression loads on the lower spine up to 7–8 times body weight [19, 20]. Recreational golfers have a higher degree of variation in muscle activation and higher maximal contraction during the golf swing.[20] Older golfers have age-related motor and skeletal system changes that influence the swing's performance [20]. With this knowledge, a standardized active warm-up for older golfers might be a solution to decrease the stress on the body [21]. However, there is no standardized, evidence-based warm-up intervention program for injury prevention yet. Integrating existing research frameworks into a practical tool is possible with The Knowledge Transfer Scheme (KTS). Therefore, we previously have developed the Golf Related Injury Prevention Program (GRIPP) intervention using the KTS without a standardized program [22].

This study aims to assess whether the GRIPP intervention effectively reduces the rates of golf-related injuries (GRI’s) in recreational golfers during 5 months. The hypothesis is that the GRIPP intervention will reduce GRI rates. The secondary outcome measures will be exposure to golf and compliance to the intervention program.

### Intervention development process

The intervention used in this protocol is developed following the KTS method which is a systematic process to develop an intervention and has been successfully applied in other sports [23–25]. The aim of the KTS is to decrease the gap between science and practice with a bottom-up approach while creating an evidence based-user friendly intervention program [22]. The most important items of the development process specifically for this study protocol are described.

The GRIPP program was developed with a focus on preventing injuries to the back and upper extremity and aimed to prevent or reduce the number of injuries. These locations were based on the distribution of injuries in scientific literature and practical experiences (most commonly seen locations by the experts) [6] (Fig. 1). The program was developed for people 45 years and older and with a handicap of 36 or lower. Because, they are the largest group (76% are 45 years or older) of representatives in golf in the Netherlands [26]. Of the registered golfers, 53.3% have a handicap of 36 or higher. The experts quantified this group as beginners/less skilled golfers and agreed that there might be other reasons for injury occurrence in this group [26].

The expert group agreed that overuse, golf posture, too much tension of muscles and wrong technique are possible causes for the occurrence injuries. Also is known, that certain golfers are playing often and others little. Therefore, it is important to monitor the exposure of the golfer and include active playing golfers with a playing frequency of at least once a week 9 holes.

During the development process of this exercise-based warm-up program the majority of the experts found the following items important:

1. Easy exercises accessible for every golfer
2. Prevention of injuries of back and upper extremity based on the distribution of injuries and expert experiences.
3. Rotation movement of the hip and spine are also important, because those are an important component of the golf swing.
4. Use of a club because it is familiar and recognizable form for the golferRecognizable exercises for the golfer related to the golf movement
5. Recognizable exercises for the golfer related to the golf movement
6. Total of six exercises

Potential facilitators mentioned were that the GRIPP intervention might positively affect the golf swing performance. Potential barriers were the use of non-practical material (such as Therabands or weights). Fear of performing, a warm-up is different from performing a golf swing and potential embarrassment when other golfers watch.

The experts suggested taking account into support, coaching and behavior change. Golfers are performing a warming-up rarely. Therefore accessibility, practical application and social interaction might be important. However, golf is an individual sport. The social interaction during golf before, during a 1.5–5 h walk and after playing, should not be underestimated. It might be an important factor in the compliance and performance of the intervention. During the pilot studies we experienced that life partners or golfers who often see each other at club activities, participated together or informed each other about the program.

The experts agreed that when golfers play a round of golf, they need to be on the tee-box approximately 10 min before their start time. With this knowledge, each warm-up session lasts approximately 5–10 min to perform. The developed program is an individual unsupervised golf athlete intervention due to the individual nature of the golf sport and reflects daily practice.

## Methods

### Study design

The GRIPP study is a two-armed cluster randomized controlled trial (RCT) with five-month of follow-ups. (NL9847 https://trialsearch.who.int). It is designed following the Standard Protocol Items: Recommendations For Interventional Trials (SPIRIT) Guideline. This statement is a checklist of recommended items to include in a clinical trial protocol [27]. The study is a cooperation between physiotherapists, sports physicians, orthopedic surgeons and research staff from Amphia Hospital, Amsterdam University Medical Centers, Erasmus University Medical Center and the Dutch Golf Federation (NGF). The intervention is a real-life setting investigation on the golf course. The need for ethical approval was waived by the Medical Review Ethics Committee of the Amsterdam Medical Centre on March 4^th^, 2021. Because the Medical Research Involving Human Subjects Act (WMO) does not apply to our study protocol. The reference number is W21-046#21.140.

### Recruitment of participants and randomization

Throughout the country, Dutch golfers (women and men, with an age of 45 years or above (year of birth 1976)) playing at a Dutch golf club will be recruited there. A top-down strategy was used to recruit the golf clubs. The Dutch Golf Federation has informed the board of the golf clubs with an informative e-mail about the study. After a positive response from the club board, further information was sent to individual members. Golfers willing to enrol are asked to read the information brochure. Before starting the study, written informed consent will be obtained from all participants with CastorEDC (CastorEDC CIWIT B.V., Amsterdam, The Netherlands). Golf clubs are randomized as clusters to minimalize contamination of individual participants [28]. Club randomization was performed based on the potential facilitators, barriers and experiences during the development process. Cluster randomization will be performed by a computer-generated scheme at the golf club level. Golf clubs are variable block randomized using random blocks of sizes 2 and 4. The coordinating investigator (SG) will hold the randomization key. The randomisation key can be revealed when group allocation is completed and definitive. Blinding of the participants is not possible due to the nature of the intervention. Blinding the coordinating researcher (SG) is impossible because the coordinating researcher corresponds with the participants during the study and is responsible for data collection.

### Study population

Participants with the following inclusion criteria are eligible for enrolment:

- Participants are golfers with a handicap of ≤ 36
- Participants are ≥ 45 years of age
- Participants play/train at least nine holes once a week (and are willing to perform the GRIPP intervention at least twice a week)
- Participants understand the Dutch language

The criterium for exclusion is not having an individual email address.

### Intervention

**Fig. 2:**
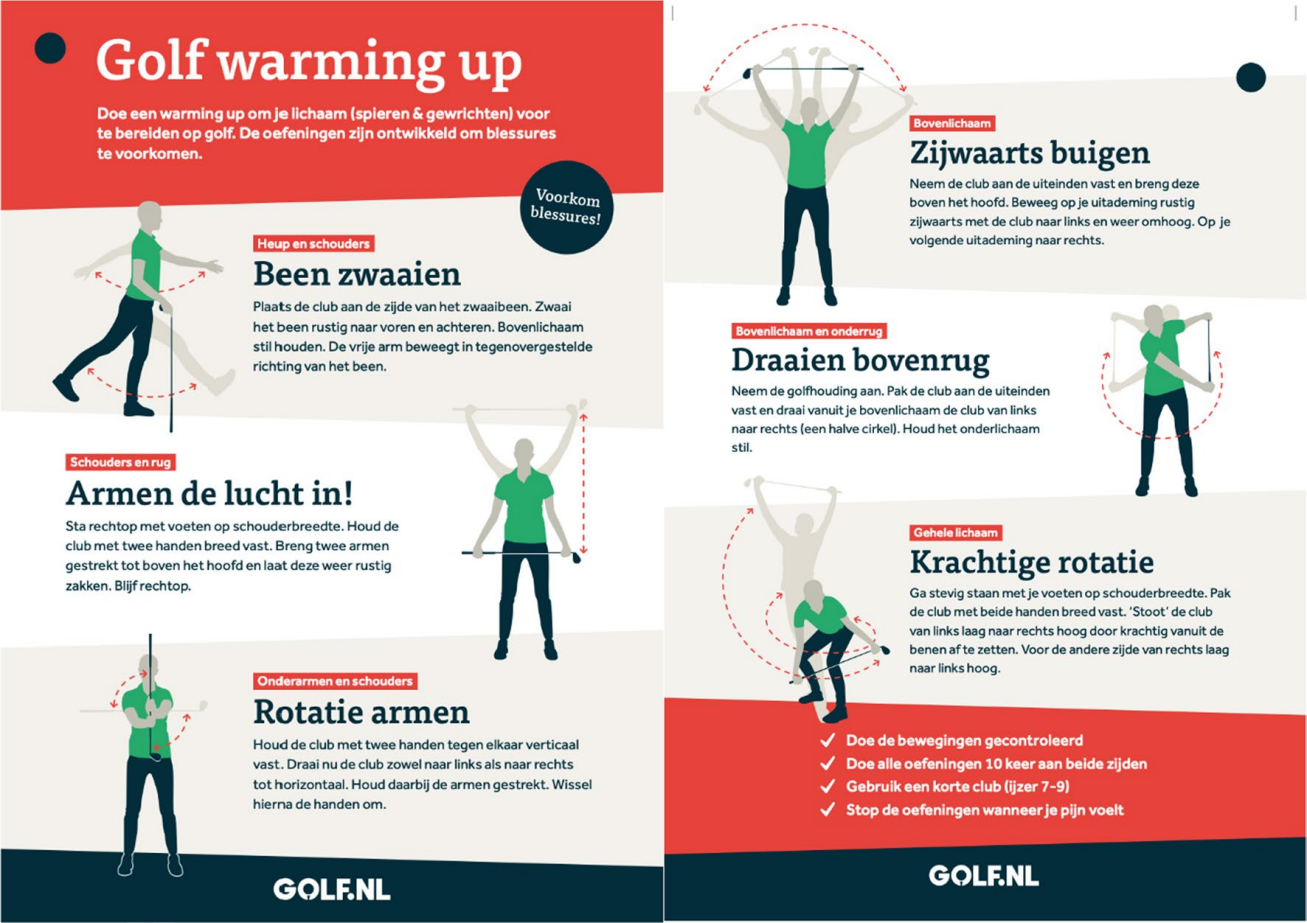
The intervention for golfers

**Fig. 3:**
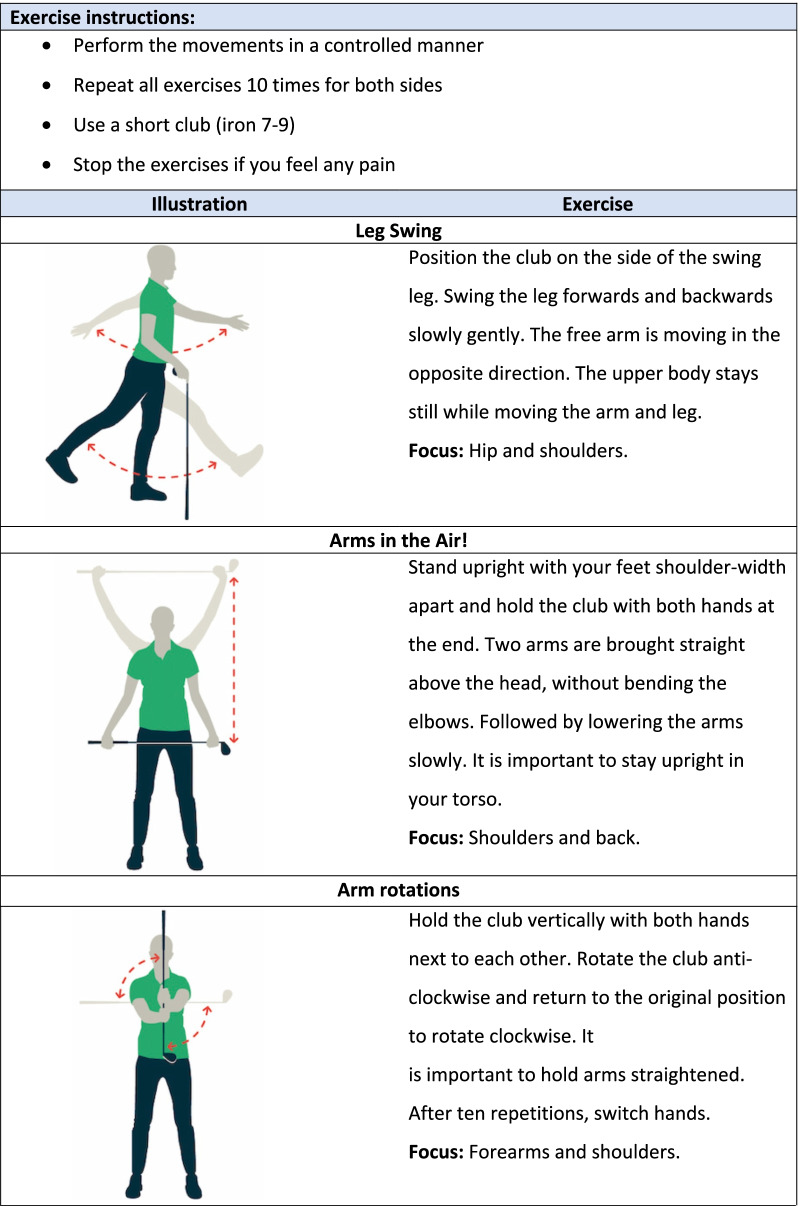

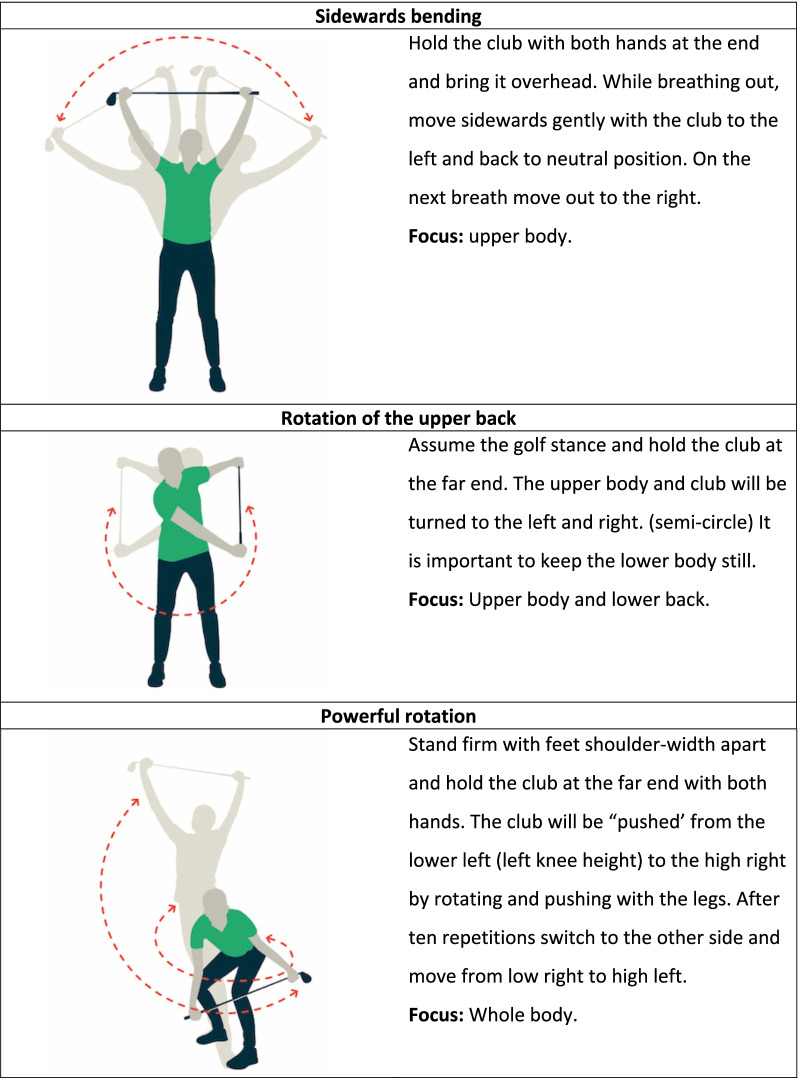
Exercises of the GRIPP intervention

Golfers allocated to the control group perform their warm-up as usual or no warm-up. Golfers allocated to the intervention group receive further instructions from the coordinating researcher (SG) about the warm-up. The intervention group is exclusively performing the GRIPP intervention. The golfer will be instructed that the warm-up is developed to prepare the body (muscles and joints) for golf and prevent injuries. Instructions will be provided through handout cards on the golf course and instruction videos digital. The handout card is presented in Fig. 2. The illustrations and the instructions for the six exercises are presented in Fig. 3. The golfer is instructed to perform all 6 selected exercises before playing or practising golf during the warm-up. For both groups, there were no limitations in playing or practising golf. The amount of practice time, holes, and days of playing golf will be questioned.

### Implementation

Instructions will be provided through handout cards on the golf course and instruction videos digital. Online instructions for the exercises will be available with a protected online link. The coordinating researcher (SG) can be approached by mail or phone if there are any questions from the golfers or clubs. The coordinating researcher (SG) can be approached by mail or phone if there are any questions from the golfers or clubs. A golfer is advised to perform a warming-up when playing golf during the 5-month intervention period. Participants in both groups are receiving no instructions on practice, play or golf lessons otherwise than performing the GRIPP intervention as a warm-up in the intervention group or in the control group performing their usual warm-up or no warm-up during the season 2021.

### Data collections/outcome measures

The baseline questionnaire consists of gender, weight, height, experience, material, previous exposure, and the golfers' current and previous health problems. Also, will be questioned at baseline preventive measures and if the golfer performs a warm-up prior the study, and if so, what it consists. Questions in the baseline questionnaire and fortnight follow-up are mostly based on the International consensus statement: methods for recording and reporting epidemiological data on injuries and illnesses in golf [29]. The definitions of injury, illness, and exposure are specified in this statement's golf-specific examples.

**Fig. 4:**
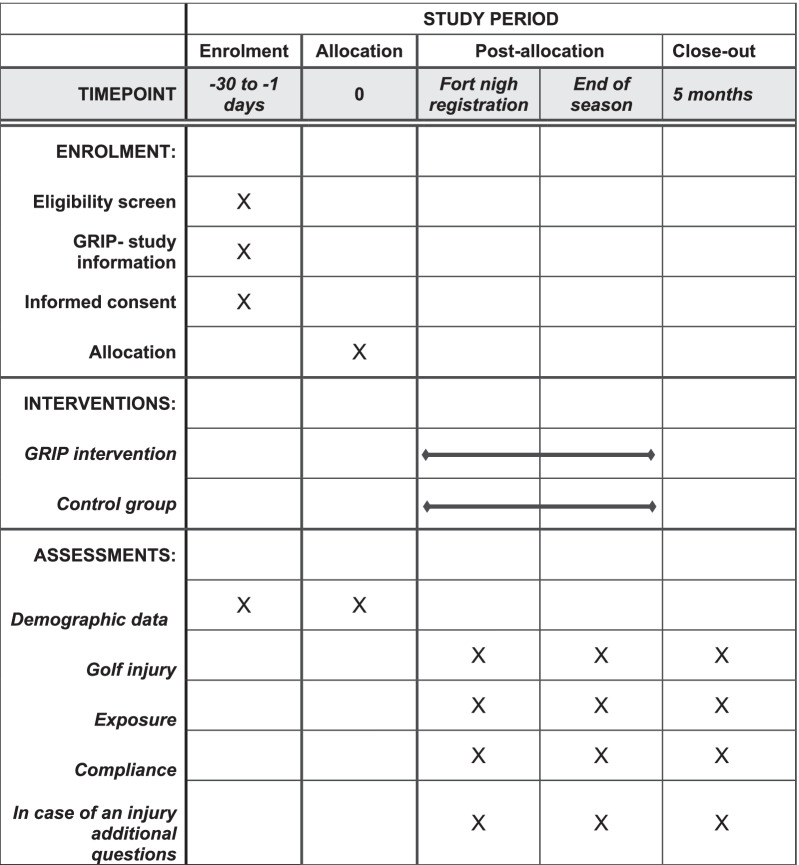
Spirit figure ( copyright following the Creative Commons “Attribution-NonCommercial-NoDerivs 3.0 Unported” license from the SPIRIT Group and adapted with Word. Source: Spirit group. Schedule Of Enrolment, Interventions, And Assessments. Available from: https://www.spirit-statement.org/schedule-of-enrolment-interventions-and-assessments/)

The current health status, exposure, and compliance to the intervention program will be questioned every fortnight with an online form. The exposure is separated into load on the golf course, driving range, putting/short game and fitness per week. This load is also further questioned with the number of days, the number of hours, how many 9 or 18 holes are played and how many balls were shot per week. The intervention group is also questioned how many times the warm-up is performed and if performed of all exercises was performed. The experience of the golf athletes will be evaluated through a structured questionnaire during the intervention period (Fig. 4).

The primary study parameter is the overall prevalence (%) of golf injuries measured with the Oslo Sports Trauma Research Center questions on health problems (OSTRC-H) every fortnight. The four key questions of the OSTRC-H on injury, illness or other health problems are related to: (1) Difficulties participating in training, practice or competition; (2) Modified training, practice or competition; (3) Influence of the problem on the performance; (4) Experienced symptoms/health complaints. Based on the four questions a severity score can be calculated. We have modified the OSTRC into a fortnight questionnaire to reduce the burden on participants because of the 5-month follow-up. This has been done previously in other studies [16, 30, 31]. The OSTRC-H collects relevant data on all health problems with this questionnaire, even those that affect the athlete but might not receive medical attention. If a health problem was reported with the four key questions, additional information will be questioned such if it was an injury or illness. Further specified into body part or what kind of illness complaints the golfer have, and if the injury was acute or overuse etc. following the golf consensus statement.

The digital questionnaires will be completed in the CastorEDC this is an electronic data capture system for clinical research trials. Other information collected during this study will be electronically saved in password-protected and secured computers.

### Sample size calculation

The sample size was calculated based on χ2 test. We considered a reduction of 40% in injury prevalence as clinically relevant. For this 40% reduction in period prevalence from 0.36 to 0.22, α of 0.05 and β of 0.80, adjusting for cluster correlations (estimated intraclass correlation of 0.05) and 11 participants per cluster, a sample of 21,17 clusters is needed [16]. Correcting for an estimated dropout rate of 30%, the sample size was set at 28 clusters (**total n = 308 approximately 2 groups of 154)**. The definition of a cluster is equal to one golf club.

### Statistical analyses

Data analysis will be performed using the Statistical Package for the Social Sciences (SPSSS). Descriptive statistics will be performed for golfers’ baseline characteristics and golf injuries of the past 12 months. For the primary outcome, a generalized linear mixed model will be used to examine the effect of the GRIPP intervention on injury prevention. The randomization location (intervention or control group) will be an independent variable. The occurrence of injury will be used as a dependent variable. Possible confounders will be analysed in the model.

## Discussion

To the best of our knowledge, there have been no previous randomized trials testing an injury prevention warm-up protocol for golfers. The GRIPP study will compare the conducted warm-up protocol with a control group who perform their warm-up as usual. Golfers of all levels are likely to benefit from the conducted warm-up protocol compared with the positive results of other effective warming-up prevention programs [16–18]. The developed program is an individual unsupervised golf athlete intervention and reflects the daily practice of predominantly unsupervised exposure of amateur golfers.

It is uncommon in an individual intervention prevention program study to cluster randomize. We developed an unsupervised program and our program reflects the daily practice. However, these unsupervised programs have problems with adherence and compliance rates [30, 32]. For example, an unsupervised intervention study advised future studies in tennis for potential improvement of adherence and exercise quality to test in a coach-based setting [30]. Based on the knowledge obtained during the development process and the reflection of daily practice, it was suggested to randomise per club, so golfers are informed and able to speak freely and practice with this warm-up. We will monitor this problem and further unsupervised exercise programs in individual sports might have advantage of our experiences of our randomization strategy.

We need to be conscious that there might be other reasons for the occurrence of injuries, such as golf professional influence and a suboptimal technique. However, it is difficult to identify this. There are presumptions that swing faults might influence the occurrence of specific golf injuries. The golfers in our study are amateur golfers of 45 years and older. These golfers likely have their own personal body limitations and injury history, and therefore each golfer has its own unique swing based on these limitations. The influence of a suboptimal swing is out of the scope of this study. It is plausible that golfers at this age and amateur level all have swing faults.

## Data Availability

Not applicable.

## Abbreviations

GRIPP: Golf Related Injury Prevention Program
GRI’s: Golf-related injuries
KTS: Knowledge Transfer Scheme
OSTRC-H: Oslo Sports Trauma Research Center questions on health problems
RCT: Randomized controlled trial
WMO: Medical Research Involving Human Subjects Act
